# Fabella Fracture as a Sentinel Sign of Underlying Anterior Cruciate Ligament Tear: A Rare Clinical Association

**DOI:** 10.7759/cureus.90340

**Published:** 2025-08-17

**Authors:** Syeda Shabistan Intekhab, Alaa Al-Taie, Syed Alam, Renan Ibrahem Adam

**Affiliations:** 1 Radiology, Hamad Medical Corporation, Doha, QAT; 2 Radiology, Hamad General Hospital, Doha, QAT; 3 College of Medicine, Qatar University, Doha, QAT; 4 Musculoskeletal Radiology, Hamad Medical Corporation, Doha, QAT

**Keywords:** acl injury, fabella fracture, musculoskeletal radiology, sesamoid bones, trauma

## Abstract

The fabella is a sesamoid bone located in the posterolateral aspect of the knee. Fabella fractures are rare and may be overlooked in patients presenting with posterolateral knee pain. The identification of a fabella fracture on radiographs may suggest underlying soft tissue injury requiring further evaluation. We report the case of a 28-year-old male patient who sustained a fabella fracture following a twisting sports-related knee injury, with magnetic resonance imaging (MRI) revealing an associated complete anterior cruciate ligament (ACL) tear. The patient was managed conservatively with physiotherapy and planned for ACL reconstruction. This case highlights the importance of fabella fractures as a potential indicator for underlying knee injuries. Early identification and advanced imaging are essential for accurate diagnosis and proper management.

## Introduction

The fabella is an inconstant sesamoid bone located within the lateral head of the gastrocnemius muscle, in the posterolateral capsule of the knee joint. It is present in approximately 10-30% of the general population and is often bilateral. It is important not to misinterpret the fabella as a loose intra-articular body on imaging [1]. Although typically asymptomatic, the fabella may be involved in various pathologies, including osteoarthritis affecting the fabella, fabella dislocation, and fabella syndrome [2].

Fabella fractures are extremely rare, with fewer than 20 cases described in the literature [3]. They are most commonly associated with high-energy knee trauma, total knee arthroplasty, or chronic mechanical stress [3]. Clinically, they typically present with posterolateral knee pain and should be considered as a rare but important differential diagnosis in patients with such symptoms [4].

The anterior cruciate ligament (ACL) is a key stabilizer of the knee joint, controlling anterior translation and rotational motion of the tibia relative to the femur. ACL tears are the most common ligamentous injuries of the knee, particularly in young patients following pivoting or high-impact sports injuries. As these injuries lead to knee instability and functional limitations, timely diagnosis and appropriate management are essential to prevent chronic sequelae [5].

This report presents a rare case of a fabella fracture following a sports-related twisting injury, which led to the identification of a complete ACL tear along with additional soft tissue injuries. The case highlights the importance of recognizing fabella fractures as potential indicators of significant underlying knee pathology.

## Case presentation

A 28-year-old male patient with no known comorbidities presented with right knee pain following a twisting injury sustained during a football game. The injury resulted in a fall, after which he was unable to continue playing due to pain. He was able to bear weight with support. On physical examination, there was swelling around the right knee joint and tenderness over the medial aspect of the knee. The patient had an antalgic gait. The anterior and posterior drawer tests were negative. The Lachman test was difficult to perform due to patient discomfort. Valgus and varus stress tests were unremarkable. The knee range of motion was painful but grossly preserved.

**Figure 1:**
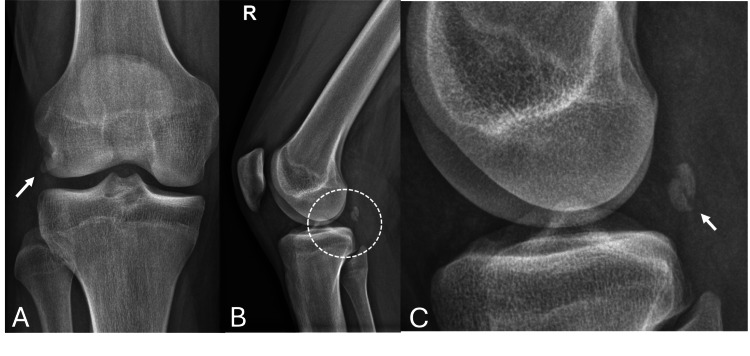
Right knee X-ray. (A) Anterioposterior view showing fabella overlapped by lateral femoral condyle (white arrow); (B) lateral view and (C) magnified image showing a fractured right fabella with minimally displaced fragments (white arrow)

An X-ray performed in the emergency department revealed a fracture of the right fabella, as shown in Figure 1.

In the emergency department, the patient was managed symptomatically with analgesics and a knee brace. He was discharged with outpatient follow-up arranged in the orthopedics department. 

**Figure 2:**
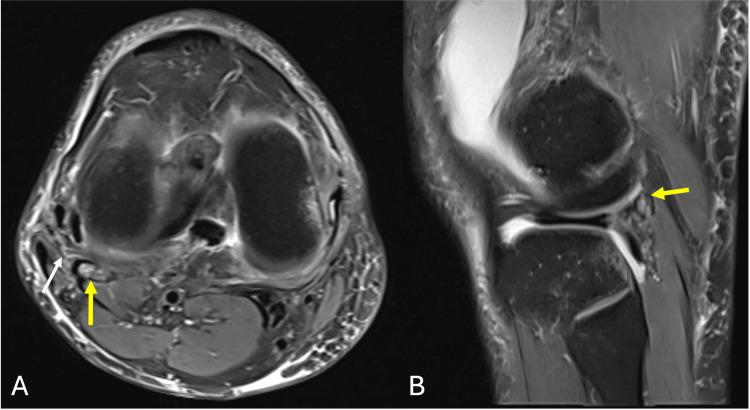
PDFS MRI (A) axial PDFS and (B) sagittal images of the right knee demonstrating bone marrow edema within the fabella, seen embedded within the lateral gastrocnemius tendon, with a visible fracture line (yellow arrows) consistent with a fabella fracture. Mild injury to the secondary stabilizers of posterolateral corner structures are also noted as shown by white arrow PDFS : proton density fat-saturated

**Figure 3:**
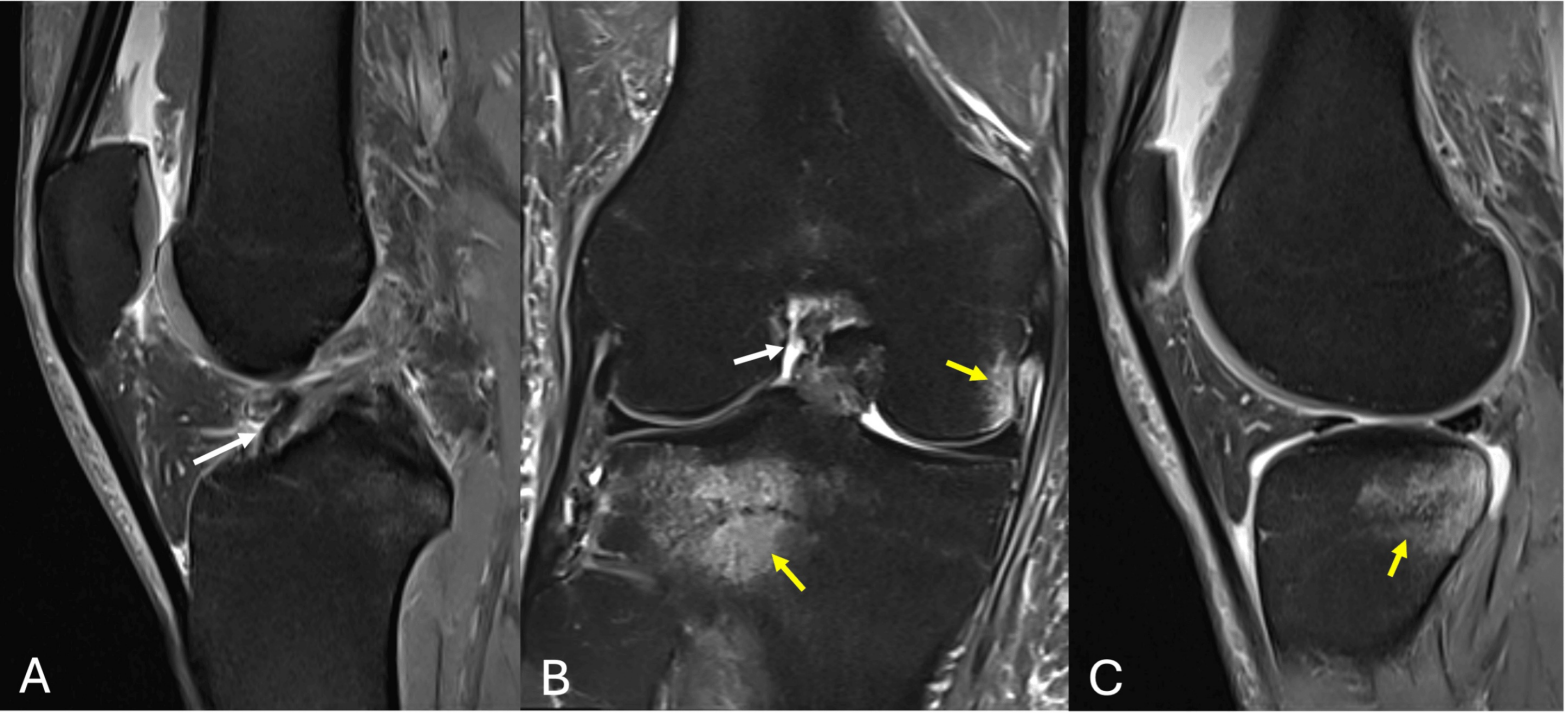
PDFS MRI (A) sagittal and (B) coronal images showing complete ACL tear from its femoral attachment (white arrows). PDFS MRI (B) coronal and (C) sagittal images showing bony contusions involving the posterolateral tibial plateau and lateral aspect of medial femoral condyle PDFS : proton density fat-saturated; ACL: anterior cruciate ligament

**Figure 4:**
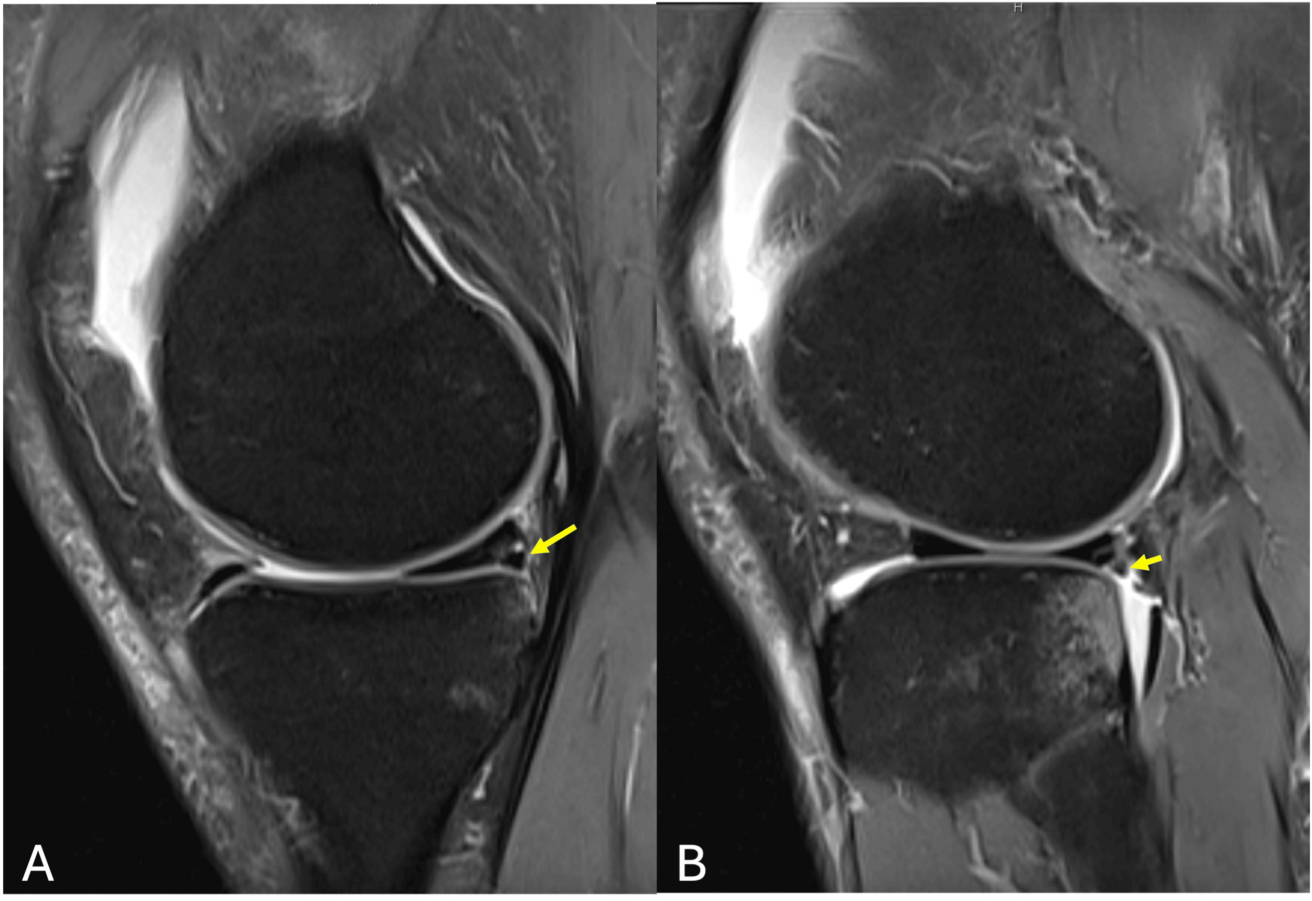
Sagittal PDFS MRI showing (A) small flap tear of posterior horn of medial meniscus and (B) small flap tear of posterior horn of lateral meniscus (yellow arrows) PDFS : proton density fat-saturated

**Figure 5:**
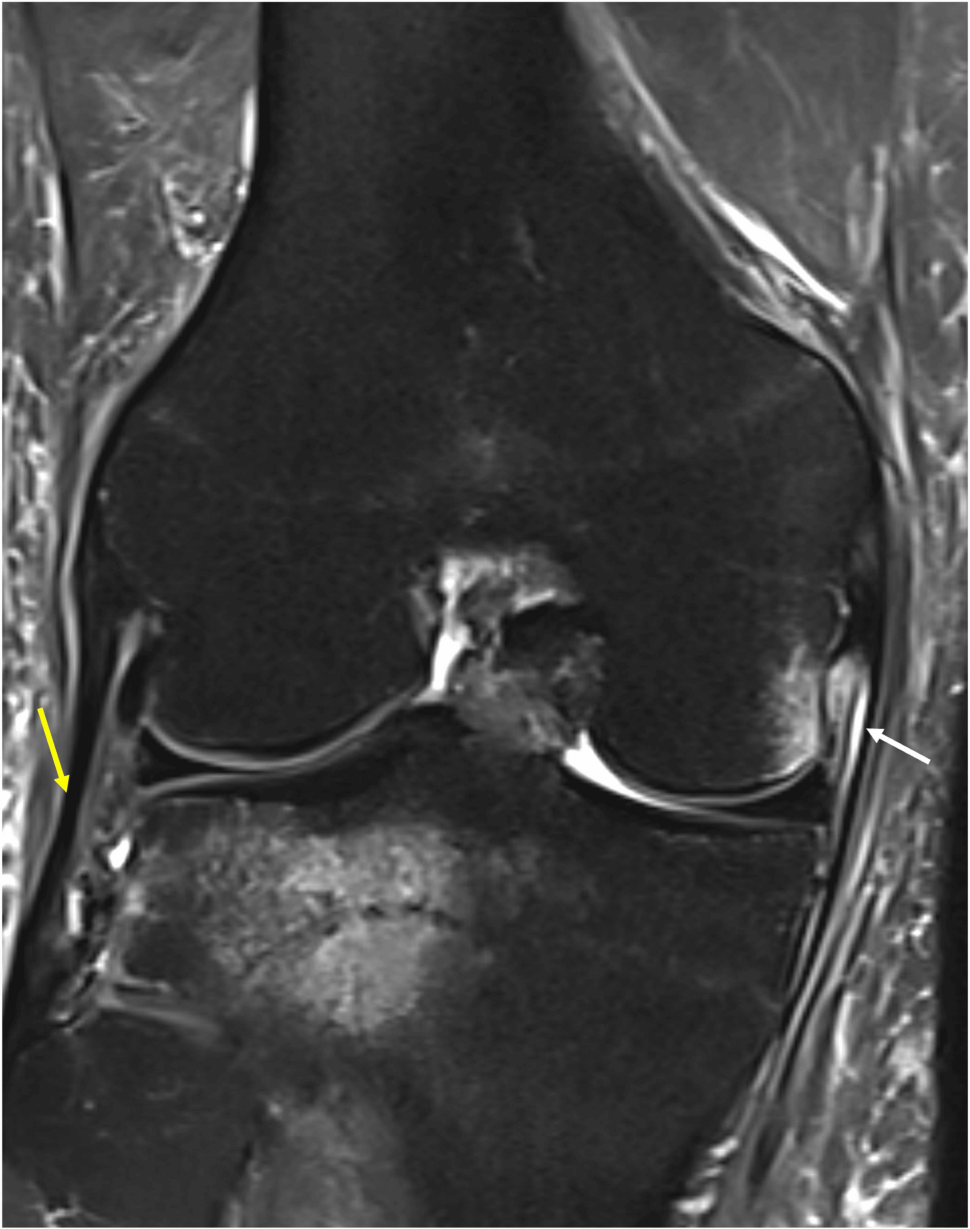
Coronal PDFS MRI showing grade 1 and 2 sprain in fibular (yellow arrow) and tibial (white arrow) collateral ligament respectively PDFS : proton density fat-saturated

Subsequent MRI of the right knee demonstrated bone marrow edema within the fabella along a distinct sagittal fracture line, consistent with a fabella fracture (Figure 2). Associated bone contusions were noted in the posterolateral tibial plateau as well as the lateral aspect of the medial femoral condyle (Figure 3). Additional findings included a complete ACL tear from its femoral attachment (Figure 3), mild injury to secondary stabilzers of the posterolateral corner (Figure 2), small flap tears of the posterior horns of both the medial and lateral menisci with associated capsular injury (Figure 4), as well as grade 1 and grade 2 sprains of the fibular and tibial collateral ligaments, respectively (Figure 5).

**Figure 6:**
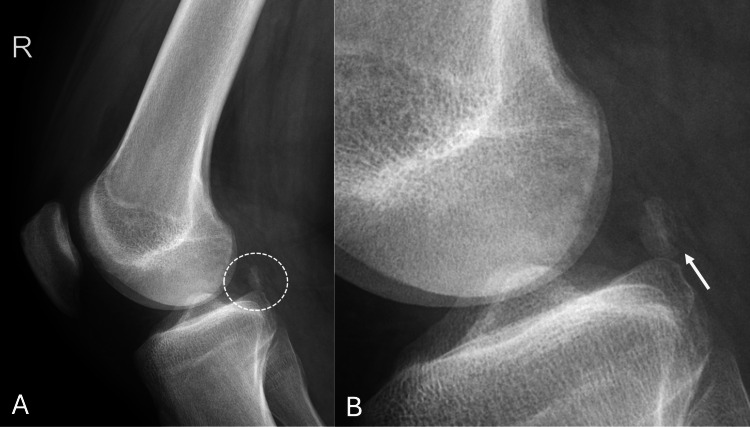
Follow up right knee X-ray (A) lateral view and (B) magnified image showing interval progressive healing of the right fabella fracture

A follow-up X-ray of the right knee, obtained 10 weeks post-injury, showed interval progressive healing of the right fabella fracture. (Figure 6).

The patient was advised to undergo physiotherapy to regain knee range of motion and quadriceps strengthening, and a plan for ACL reconstruction in the future. The patient's knee pain and swelling improved gradually with physiotherapy. At the five-month follow-up appointment, there were no clinical signs of instability. Patient was advised to continue physiotherapy and with a plan for arthroscopic ACL reconstruction surgery. 

## Discussion

The fabella is an inconstant sesamoid bone located within the posterolateral capsule of the knee joint, embedded in the lateral head of the gastrocnemius muscle. It articulates with the posterior articular surface of the lateral femoral condyle [4]. It is located at a critical intersecting point of tensile forces from the lateral head of the gastrocnemius tendon, the arcuate ligament, the fabellofibular ligament, and the oblique popliteal ligament. Therefore, the fabella functions analogously to the patella, aiding in the transmission and stabilization of flexion forces across the posterior knee [6].

The posterolateral corner (PLC) of the knee is a complex anatomical and functional region comprising both primary and secondary stabilisers. It plays a crucial role in resisting varus stress, external tibial rotation, and posterior translation of the tibia. The three major stabilisers of the PLC include the fibular collateral ligament, the popliteus tendon, and the popliteofibular ligament. The fabellofibular ligament, a secondary stabilizer, originates from the fibella and inserts into the fibular head. Consequently, a fracture of the fabella may compromise the structural integrity of this stabilising complex [6,7]. 

Fracture of the fabella is a rare entity that typically presents with posterolateral knee pain. Among the cases reported in the literature, common etiologies include direct trauma, high-energy mechanisms such as motor vehicular accidents, and postoperative complications following total knee arthroplasty [3,8]. Dashefsky et al. [9] and Marks et al. [10] described cases in which the fabella fracture occurred due to twisting injuries and direct blows to the knee during football, similar to the mechanism observed in our patient [9,10].

In most cases, plain radiography is sufficient for diagnosis, typically revealing a distinct fracture line and, occasionally, displaced bone fragments in the posterolateral aspect of the knee [3]. The fabella is more clearly visualised in the lateral view. As the fabella is often present bilaterally, comparing radiographs of the contralateral knee can be useful in confirming the diagnosis [11]. Notably, nearly all previously reported cases [3,11-16] demonstrated a transverse fracture line, except for one [12]. In contrast, our case revealed a vertically oriented fracture line, making it an uncommon presentation.

Although computed tomography (CT) or MRI is not routinely required for diagnosing fabella fractures, advanced imaging may be warranted in case of high-energy trauma to assess for associated injuries to the cruciate ligaments and posterolateral corner structures. In fact, the identification of a fabella fracture on a plain radiograph should raise suspicion of concomitant soft tissue injuries and prompt evaluation with MRI or CT to ensure early diagnosis and appropriate management [11,15].

MRI was performed in three of the fabella fracture cases reported in the literature [3,11,14]. Barreto et al. described a post-traumatic fabella fracture in which MRI revealed bone marrow edema in the fabella and gastrocnemius muscle, along with bony contusions in the femoral condyles, tibial plateau, and the fibular head; no meniscal or ligamentous injuries were identified [11]. Zhou et al. described a case demonstrating contusions of the tibial plateau [14]. Buruian et al. described a case of fabella fracture following a motor vehicle accident, where bilateral knee MRI revealed bone marrow oedema of the tibial plateau and distal femur on the side of the fracture, without additional meniscal or ligamentous injuries. However, the contralateral knee showed posterolateral corner injury, complete rupture of both cruciate ligaments, and menisco-capsular injury of the posterior horn of the medial meniscus [3].

The most distinctive MRI finding in our case was the atypical pattern of bone contusions, involving the posterolateral tibial plateau and the lateral aspect of the medial femoral condyle, which differs from the pattern reported in previous cases. Additionally, MRI revealed a complete ACL tear, mild injury to the posterolateral corner, and menisco-capsular injuries involving the posterior horns of both the medial and lateral menisci. These findings highlight the importance of advanced imaging in fabella fractures to assess for any associated soft tissue injuries. Identifying these injuries early is critical, as they may require further management and could lead to long-term morbidity if missed [3,14,16].

To date, no prior case reports have described menisco-ligamentous injuries associated with fabella fracture. However, posterolateral corner injuries are frequently associated with cruciate ligament tears [6], as was evident in our case. Additional case reports with detailed MRI findings are needed to better characterize the pattern of bone contusion and associated soft tissue injuries in patients with fabella fractures. 

Most reported cases of fabella fractures have been successfully managed with conservative treatment. Only two cases in the literature required surgical intervention [9,15]. However, surgical management and physiotherapy may be necessary in cases with associated ligamentous injuries, depending on the severity and functional instability [11]. In our case, the fabella fracture demonstrated progressive interval healing with conservative management along with improvement in the patient's symptoms. The patient was, however, scheduled for reconstruction surgery for an associated complete ACL tear. 

## Conclusions

Fabella fractures are rare and often overlooked causes of posterolateral knee pain. This case report highlights the need for clinicians and radiologists to maintain a high index of suspicion for fabella fractures, especially in patients presenting with posterolateral knee pain following high-energy knee trauma. Their presence should prompt a thorough evaluation for associated injuries, particularly to the ACL. Recognizing these injuries early can help avoid misdiagnosis and lead to better patient outcomes. Therefore, fabella fractures are not only rare isolated findings but also potential indicators of underlying knee instability.
